# Physical activity reduces cardiovascular risk and mortality in people with severe mental illness: a cohort study using accelerometry

**DOI:** 10.1192/j.eurpsy.2024.164

**Published:** 2024-08-27

**Authors:** Y. Y. Liang, J. Du, Y. Zhou

**Affiliations:** Center for Sleep and Circadian Medicine, The Affiliated Brain Hospital of Guangzhou Medical University, Guangzhou, China

## Abstract

**Introduction:**

Cardiovascular disease (CVD) is a leading cause of excess mortality in people with severe mental illness (SMI). Physical activity (PA) is widely acknowledged with multiple health benefits, but associations of PA with incident CVD, all-cause and CVD mortality in people with SMI remain unclear.

**Objectives:**

To determine dose-response and intensity-specific associations of PA with incident CVD, all-cause and CVD mortality in people with SMI.

**Methods:**

This prospective cohort study was conducted on 6313 SMI participants with accelerometry data from UK Biobank (mean age 61.05 years) from February 2013 to November 2021 (median 7-year follow-up). Moderate-to-vigorous PA (MVPA) was categorized by meeting the guideline level or not, while total PA and light-intensity PA (LPA) were grouped by tertiles. Incident CVD, all-cause and CVD mortality ascertained by hospital and death registries were main outcomes.

**Results:**

PA was inversely associated with the risk for incident CVD (P_overall_ < 0.05 for total PA and MVPA, P_nonlinearity_ > 0.05 for all PA), all-cause mortality (P_overall_ < 0.05 for all PA, P_nonlinearity_ < 0.05 for total PA and LPA), and CVD mortality (P_overall_ < 0.001 for total PA and LPA, P_nonlinearity_ < 0.05 for all PA). Performing guideline-recommended volume of MVPA was associated with a reduced risk of 19% for incident CVD (95% CI, 0.67-0.98), 42% for all-cause mortality (95% CI, 0.43-0.79), and 50% for CVD mortality (95% CI, 0.31-0.82). A combination of recommended MVPA and a moderate volume of LPA was associated with the lowest risk, mitigating 21% risk for incident CVD, 59% for all-cause mortality, and 78% for CVD mortality.

**Conclusions:**

Primary engagement of guideline-recommended MVPA, supplemented with moderate amount of LPA, was associated with lower risks for incident CVD, all-cause and CVD mortality among people with SMI.

**Acknowledgements:**

This research has been conducted using the UK Biobank Resource under Application Number 58082.

**Funding Support:**

This work was supported by the National Natural Science Foundation of China (grant number 32100880), Guangzhou Municipal Key Discipline in Medicine (2021-2023), Guangzhou High-level Clinical Key Specialty, and Guangzhou Research-oriented Hospital. The funders had no role in the design and conduct of the study; collection, management, analysis, and interpretation of the data; preparation, review, or approval of the manuscript; and decision to submit the manuscript for publication.

**Disclosure of Interest:**

None Declared

